# Synopsis of Human Anatomy

**Published:** 1889-08

**Authors:** 


SYNOPSIS OF HUMAN ANATOMY, being a complete compound of anatomy, including the anatomy of the viscera and numerous tables by James K. Young, M. D., Instructor in orthopedic surgery and assistant demonstrator of surgery in the University of Pennsylvania, attending orthopœdic surgeon, out patient department, University Hospital; Fellow of the College of Physicians, etc. etc. Philadelphia and London. F. A. Davis, publisher, 1889.

The above is a plain, concise and complete synopsis of human anatomy, and admirably adapted, both in its matter and illustrations, to the wants of the student, and as a work of reference to the surgeon, the teacher, and to the practitioner.

A DOCTOR'S POCKET KNIFE.—We are in receipt of a beautiful and very convenient Doctor's knife kindly sent us by the Western Supply Co., of Ohio. It answers every purpose of an ordinary pocket knife, and contains also a small spatula, useful for dosing out powders or spreading a salve, a gum lancet and a small curetle useful for extracting a foreign body from the nose and other purposes It is not too bulky in size, and contains a transparent handle beneath which the name of the owner may be printed. To any one sending us two new cash subscribers ($4.00) we will have mailed to him one of these beau. tiful knives with his name engraved on the handle. The retail price of this doctor's knife is $2.25.

